# Peroral endoscopic myotomy using a novel thin therapeutic scope

**DOI:** 10.1055/a-2334-1024

**Published:** 2024-06-18

**Authors:** Hiroshi Tanabe, Hiroya Sakaguchi, Hirofumi Abe, Hitomi Hori, Chise Ueda, Shinwa Tanaka, Yuzo Kodama

**Affiliations:** 1 Division of Gastroenterology, Department of Internal Medicine, Kobe University Graduate School of Medicine, Kobe, Japan

Peroral endoscopic myotomy (POEM) has been gaining in popularity as an effective minimally invasive treatment for achalasia 1 . However, submucosal fibrosis and thicker esophageal mucosa in patients with achalasia can complicate scope insertion into the submucosa. Furthermore, passing the scope through the esophago-gastric junction can be challenging when the lower esophageal sphincter (LES) is tight. A thinner therapeutic scope may be desirable for such challenging POEM-based procedures. POEM with a nasoendoscope is a potential solution for such situations and has shown short-term outcomes comparable to those of conventional POEM 2 . However, only certain types of endoknives can be passed through the smaller working channels of nasoendoscopes, and a lack of scope stiffness may make their manipulation difficult. A novel thin therapeutic endoscope (EG-840TP; Fujifilm Co., Tokyo, Japan), with a diameter of 7.9 mm, a wide (3.2-mm) working channel, a wide-ranging downward angle of 160°, and enhanced stiffness compared to a nasoendoscope may overcome these challenges ( Fig. 1 ).

**Fig. 1:**
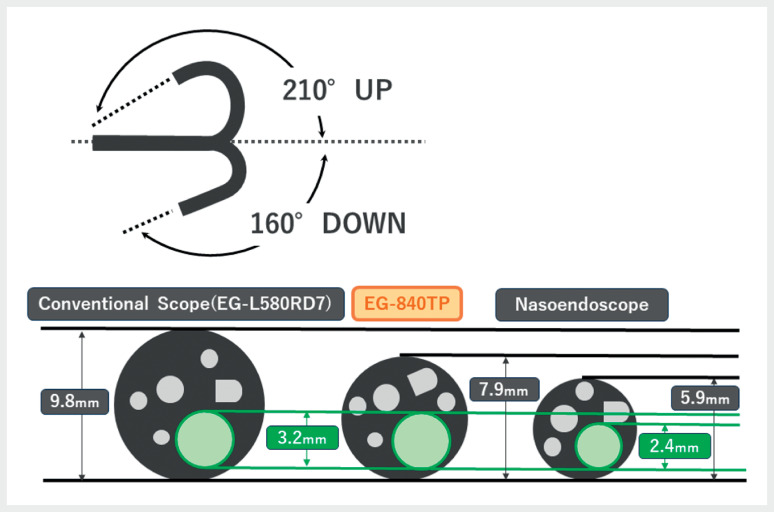
The EG-840TP – with a diameter of 7.9 mm, a working channel of 3.2 mm, a downward angle of 160°, and enhanced stiffness compared to nasoendoscopes – is well-suited for peroral endoscopic myotomy procedures.

A 28-year-old woman with achalasia (Chicago Classification type 1, Grade II dilation) was referred to our hospital, where we performed POEM using a EG-840TP scope ( Video 1 ).

Peroral endoscopic myotomy (POEM) using a novel thin endoscope (EG-840TP).Video 1

An approach perpendicular to the esophageal wall is typically preferred for easy insertion into the submucosa during POEM. Unlike conventional therapeutic endoscopes with narrow-ranging downward angles, the EG-840TP facilitated scope insertion without the need for additional dissection of the entry site ( Fig. 2 ). Its thinner tip resulted in a smaller entry and streamlined closure using clips ( Fig. 3 ). Although creating a submucosal tunnel was challenging, owing to the limited workspace available as the patient had a tight LES, the smaller EG-840TP allowed us to create a sufficient tunnel ( Fig. 4 ).

**Fig. 2:**
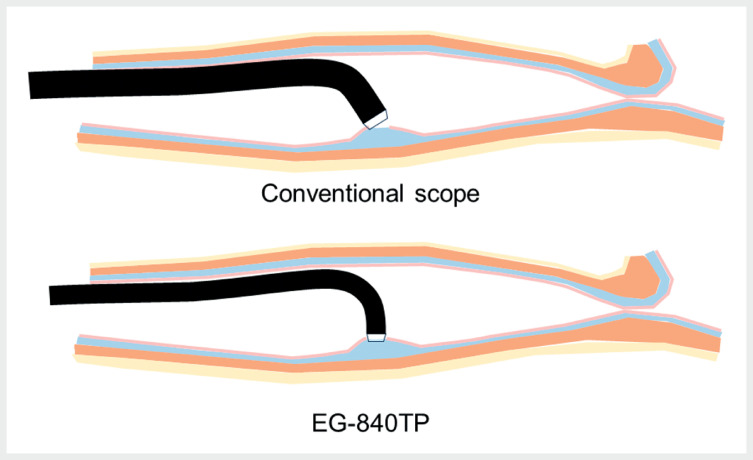
**a** Conventional endoscopes with 120° downward angles lack sufficient force transmission in the narrow esophagus, making vertical approaches challenging. **b** The EG-840TPʼs large downward angle facilitates easy scope insertion into the submucosa using a downward angle.

**Fig. 3:**
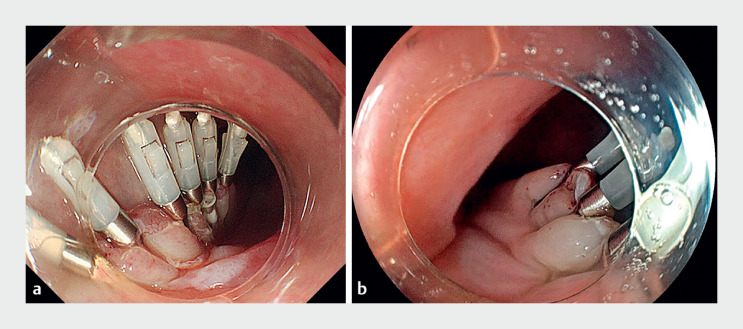
**a** Closure with clips is performed using a conventional endoscope. The prolonged incision requires a significant number of clips. **b** Closure with clips is performed using the EG-840TP. The slim endoscope diameter results in a shorter entry incision length, facilitating easy closure with fewer clips.

**Fig. 4:**
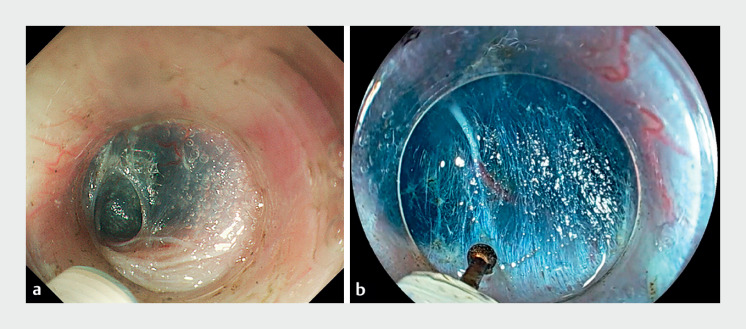
**a** Submucosal tunneling near the lower esophageal sphincter (LES) is performed using a conventional endoscope. **b** Submucosal tunneling near the LES is performed using an EG-840TP. The novel scope makes it easier to secure the workspace, simplifying the creation of the submucosal tunnel.

This novel endoscope, with its potential advantages in challenging circumstances, may represent a new standard therapeutic endoscope for POEM procedures.

Endoscopy_UCTN_Code_TTT_1AO_2AP

## Notes

Endoscopy E-Videos https://eref.thieme.de/e-videos
*E-Videos* is an open access online section of the journal *Endoscopy* , reporting on interesting cases and new techniques in gastroenterological endoscopy. All papers include a high-quality video and are published with a Creative Commons CC-BY license. Endoscopy E-Videos qualify for HINARI discounts and waivers and eligibility is automatically checked during the submission process. We grant 100% waivers to articles whose corresponding authors are based in Group A countries and 50% waivers to those who are based in Group B countries as classified by Research4Life (see: https://www.research4life.org/access/eligibility/ ). This section has its own submission website at https://mc.manuscriptcentral.com/e-videos .
